# A minimal model for a slow pacemaking neuron

**DOI:** 10.1186/1471-2202-13-S1-P101

**Published:** 2012-07-16

**Authors:** Alexey Kuznetsov, Denis Zakharov

**Affiliations:** 1 Department of Mathematical Sciences and Center for Mathematical Biosciences, Indiana University Purdue University Indianapolis, Indianapolis, IN 46202, USA; 2 Nonlinear dynamics department, Institute of Applied Physics, RAS, Nizhny Novgorod 603950, Russia

## 

We have constructed a phenomenological model for slow pacemaking neurons. These are neurons that generate very regular periodic oscillations of the membrane potential. The examples of these neurons are serotonin-containing neurons from the raphe nuclei, noradrenergic neurons located in the pontine nucleus locus coeruleus (LC) and dopaminergic neurons from the substantia nigra pars compacta. Many of these neurons also differentially respond to various types of stimulation. In particular, stimulation by injecting a current into the cell body (applied somatic depolarization) is expected to elicit bursting similar to stimulation by inputs from other neurons (synaptic currents), but it does not.

**Figure 1:**
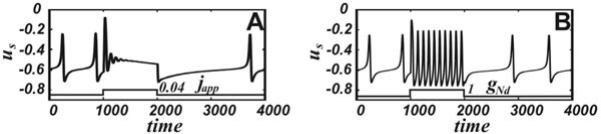
The NMDAR activation elevates the frequency, while the applied depolarization blocks oscillations. The voltage time-series are shown in response to square pulses of the applied depolarization (A) and NMDAR activation (B).

We have separated the most important property of the neuron that allows for the differential responses (see Fig. 1). Our model is based on FitzHugh-Nagumo (FHN) oscillator and implements a nonlinearity introduced by a current that depends on an ion concentration. The nonlinearity is crucial for such differentiation. We have explicitly shown that when the nonlinear activation function is replaced by a linear dependence, the model responds similarly to all stimuli and shows very little frequency variation. These are the properties of the classical FHN oscillator. Thus, the new nonlinear dependence allows for differentiating responses to various stimuli.

In the DA neuron, an SK-type Ca2+-dependent K+ current provides the necessary nonlinearity. Presumably, the same current works in serotonergic neurons. No data is available for other neurons, and the SK current may not be the only current that differentiates the responses. The mechanism works for other currents with a sigmoidal activation function. In fact, any function that starts flat and then sharply increases its slope works. The saturation part of the sigmoidal dependence is not necessary for the frequency responses. The current may depend on ion concentration, on the voltage, or on both variables. This further expands the applicability of our results to neurons expressing various currents. Therefore, in a wide class of neurons, the nonlinearity of a conductance will cause distinct responses to stimuli.

